# A Novel Nickel-Plated Carbon Fiber Insert in Aluminum Joints with Thermoplastic ABS Polymer or Stainless Steel

**DOI:** 10.3390/ma16175777

**Published:** 2023-08-23

**Authors:** Yoshitake Nishi, Kouhei Sagawa, Michael C. Faudree, Helmut Takahiro Uchida, Masae Kanda, Satoru Kaneko, Michelle Salvia, Yoshihito Matsumura, Hideki Kimura

**Affiliations:** 1 Faculty of Emeritus, Tokai University, Hiratsuka 259-1292, Japan; west@tsc.u-tokai.ac.jp; 2 Graduate School of Science & Technology, Tokai University, Hiratsuka 259-1292, Japan; helmutuchida@tokai.ac.jp (H.T.U.); kanda@isc.chubu.ac.jp (M.K.); ncc1701d@keyaki.cc.u-tokai.ac.jp (Y.M.); kimura@tokai-u.jp (H.K.); 3 Graduate School of Engineering, Tokai University, Hiratsuka 259-1292, Japan; sagawa.kouhei@tokai.ac.jp; 4 Kanagawa Institute of Industrial Science and Technology (KISTEC), Ebina 243-0435, Japan; satoru@kistec.jp; 5 Ecole Centrale de Lyon, CEDEX, 69134 Ecully, France; michelle.salvia@ec-lyon.fr; 6 Faculty of Liberal Arts and Science, Tokyo City University, Yokohama 224-8551, Japan

**Keywords:** Al, joint, carbon fiber, Ni-plating, stainless steel, ABS, composites, specific strength, resistance energy

## Abstract

New types of hybrid aluminum joints: Al-acrylonitrile butadiene styrene (ABS) carbon fiber reinforced thermoplastic polymer (CFRTP) designated Al/**Ni**-CFP/ABS, and Al-18-8 Stainless steel, Al/**Ni**-CFP/18-8, by Ni-plated carbon fiber plug (Ni-CFP) insert not before seen in the literature have been fabricated. The goal is to take advantage of extremely high ~6 mm CF surface area for high adhesion, to enhance the safety level of aircraft and other parts. This is without fasteners, chemical treatment, or glue. First, the CFP is plated with Ni. Second, the higher melting point half-length is spot welded to the CFP; and third, the remaining half-length is fabricated. The ultimate tensile strength (UTS) of Al/**Ni**-CFP/ABS was raised 15 times over that of Al/ABS. Normalized ^c^UTS according to CFP cross-section by Rule of Mixtures for ^c^Al/**Ni**-CFP/18-8 was raised over that of ^c^Al/**Ni**-CFP/18-8 from 140 to 360 MPa. Resistance energy to tensile deformation, *U*_T_, was raised 12 times from Al/ABS to Al/**Ni**-CFP/ABS, and 6 times from Al/CFP/18-8 to Al/**Ni**-CFP/18-8. Spot welding allows rapid melting followed by rapid solidification for amorphous metal structures minimizing grain boundaries. The Ni-coating lowers or counters the effects of brittle Al_4_C_3_ and Fe_x_C formation at the interface and prevents damage by impingement to CFs, allowing joints to take on more of the load.

## 1. Introduction

Aluminum is an extremely versatile metal that has been applied to numerous articles in aerospace, architecture, fashion, and decoration of various articles [1]. Joining technologies have been advancing for commercial aircraft, vehicles for space travel, building construction, wind turbines, and sporting goods, among numerous others. Demand for lightweight materials such as Al alloys, polymers, and their composites [2,3,4] has been growing with the advantages of being lightweight with high structural strength for environmental friendliness [5].

Al has been a material of choice requiring joining for lightweight vehicle applications due to its many attractive properties, including high electrical and thermal conductivity, low specific weight, and shiny silver color. It also has strong resistance to corrosion by passivation from its affinity to oxygen forming a protective oxide layer. Al has become an essential material utilized for aerospace and other technologies due to its lightweightness and high specific strength. Al is inexpensive (US $2.42 per kg; 22 February 2023) [6] as the third most abundant element in Earth’s crust, and is recyclable being able to be remelted and reformed apparently indefinitely without losing its quality. Moreover, Al has higher process deformability than steels.

**Figure 1:**
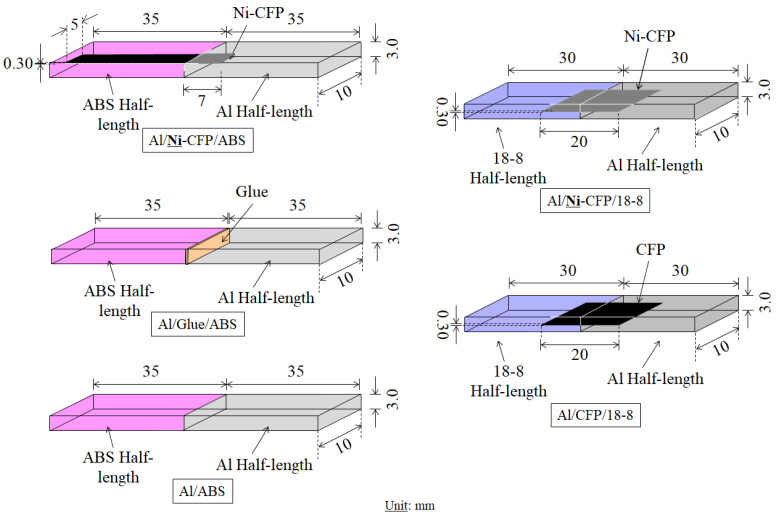
Schematic illustrations of Al/ABS joints: Al/**Ni**-CFP/ABS, Al/Glue/ABS, and Al/ABS and Al/18-8 joints: Al/**Ni**-CFP/18-8 and Al/CFP/18-8 for tensile testing [7,8].

The goal is to implement a nickel coated cross-weave carbon fiber plug (Ni-CFP) to join two widely used engineering materials with Al: (1) acrylonitrile butadiene styrene (ABS) thermoplastic (TP) polymer to construct Al/**Ni**-CFP/ABS joints [7]; and (S2) 18-8 (~18 wt.% chromium, ~8 wt.% nickel) stainless steel to construct Al/**Ni**-CFP/18-8 joints [8]. This is without the use of fasteners, chemical treatments, or catalysts. Figure 1 shows schematic illustrations of Al/ABS and Al/18-8 joint tensile testing specimens [7,8]. 

The purpose of the novel Ni-CFP is to take advantage of the high contact surface area of the 6 mm diameter CF. Moreover, the Ni-coating is applied because it acts as a barrier preventing the encroachment of CF, along with lowering or countering effects of brittle carbide formation during welding, such as Al_4_C_3_ in the case of Al [7], and FeC, Fe_2_C, Fe_3_C, Cr_2_C in the case of 18-8 [8]. The Ni also promotes mutual diffusion between Ni and the metal as a gradient, to take on part of the load to strengthen the joint during deformation [7,8]. Spot welding by electron beam is employed since it allows two different materials with different melting points (MPs) (e.g., Al and 18-8) to be welded to the CFP separately to overcome the MP difference. For Al-ABS joints, Al and ABS polymer are affixed to the CFP separately, hence the fabrication process here is referred to as a “partial” welding method with rapid melting and solidification [8]. MPs of Al and 18-8 are reported to be 660 °C [9] and 1400–1450 °C [10], respectively. ABS, on the other hand, is amorphous and does not have a MP: it softens quite rapidly in the window above its *T*_g_ (107 °Cs) [11] to the Vicat softening temperature (111 to 112 °C) [12].

An advantageous material for joining with Al is TP polymer since, like Al, can be remelted and reformed for recyclability. Since ABS is a low cost, widely used TP with high crack and impact resistance, it is attractive to join with Al for strong light-weight applications. The high impact strength of ABS is wrought out by its polymer blend consisting of poly-butadiene (PB) elastomer with acrylonitrile styrene (AS), with high rigidity, and stiffness [13,14,15,16].

ABS has a low softening temperature range of 107 °C to 112 °C [11,12] which makes it practical for injection molding and 3-D printing and is used for computer keyboards, LEGO toys, luggage, binoculars, and many household goods.

Austenitic 18-8 stainless steel [10] is commonly joined with Al [17,18,19,20,21,22] for many applications due to its exceptional strength, temperature oxidation resistance, and electrical and thermal resistivity [14]. Al and 18-8 have high resistance to corrosion at ambient temperature and can be easily recycled by re-melting and solidification.

Traditionally, fasteners, bolts or rivets have been used in making joints, however, disadvantages can include damage by hole drilling, stresses around the hole, decrease in fatigue strength, and fasteners themselves increasing the weight, hence, it is desirable to construct a fastenerless joint. The strength of joints of widely-used fastenerless fabrication procedures of brazing or welding are shown to be insufficient due to the formation of brittle chemical compounds [8]. For example, 18-8 cannot be joined well with pure Al by welding from the formation of a brittle phase at the junction [8]. When alloying steel and Al, undesirable Fe_3_Al is formed acting as crack generation sites, the small cracks being below 1 μm are taken above the critical at high temperature of welding, therefore conventional welding methods cannot be applied. Moreover, utilizing large radiative heat, it is challenging to weld an exceedingly thermally conductive metal (Al), with that of high thermal resistance (18-8).

To control brittle compound formation by melting for Al/Stainless steel joints, however, several types of solid joining methods with metallic bonds have been proposed in the literature [19,20,21,22]. A friction-bonded joint of low-carbon steel with Al-Mg alloy was developed with 306 MPa maximum strength [19]. A plastic flow joint by forming a projection of stainless steel to Al alloy at Al-softening temperature was fabricated for use as sensors for automobile parts [20]. Also, Nippon Steel has achieved a semi-hot roll process for Al clad coils to be joined with either stainless steels or Ti for use in induction cooking heaters [21,22].

A popular joining method with a proven track record has been rapid melting prior to rapid solidification. Several methods are reported including electron beam [23,24,25], laser [26], direct current [27,28], alternating current [29], and inductive magnetic field [30]. A spot beam is used in this study since it has the benefits of a strong focused beam, accurately controlled energy for achieving rapid melting before the rapid solidification, and vacuum conditions preventing intrusion into the molten metal by oxides and nitrides in the atmosphere. Spot beam has been used to fabricate Metal/Metal and Metal/Polymer joints utilizing a Ni-plated CFP [7,8] taking advantage of the rapid melting-rapid solidification process.

To weld, the cross-weave CFP is first simply placed into a formed gap in the metals. 

Although stress concentrators can be generated by spaces in the weave, flaw sensitivity is reduced by the weave pattern itself substantially increasing strength of composites [31]. But the drawbacks of CF surface are inertness by sparse functional groups, negligible wettability, and interfacial chemical instability hindering mechanical strength when coupled with Fe or other metals [32,33]. A study of CFRAl samples by high-resolution transmission electron microscopy (HRTEM) with electron spectroscopy for chemical analysis (ESCA), found undesirable Al_4_C_3_ nucleates heterogeneously from CF and grows towards the Al matrix with lath like crystals after heat treatment lowering mechanical properties of the composite [34]. 

It is difficult to wet the CF with molten Al: contact angle between molten Al and graphite at 1023K is reported to be nearly 140° [34,35]. Titration experiments found contact angle decreased with increasing temperature; however, above 1273K interfacial Al-C reaction resulted in brittle Al_4_C_3_ phase formation [34] which severely damages the CF [36].

To remedy this, CFs have been coated with metals including Cu, Ag, Co, Cr, Fe, Ti and Ni [32,37,38,39] by physical vapor deposition (PVD), chemical vapor deposition (CVD), sol-gel, electroless plating, and electroplating [40,41,42,43,44]. Ni coating to CF is reported to lower the contact angle from 140° to just 4° [45] hence several methods of coating CF with Ni are noted here. Wire-mesh catalysis has been found to result in a Ni coating that is uniform and compact, the quality being highly contingent on the catalysis process [46]. Carbonyl metal organic chemical vapor deposition (MOCVD) using a precursor of Ni(CO) has been utilized to deposit pure Ni onto CFs with heightened adhesion, raising fracture strength by 34.9%, with the added benefit of enhanced resistance to oxidation more than uncoated CFs [47]. In addition, electroplating techniques to coat Ni onto CF have shown success [35,48,49]. For unidirectional CF-Al matrix composites (CFRAl), electroless plating of Ni to CFs increased bending strength and interlaminar shear strength (ILSS) by 81% and 86%, respectively [35]. Moreover, electroless plating method has been found to raise the tensile stress of CF-metal matrix composites (CF-MMC) with decent bonding and uniform Ni distribution on CFs, although tensile stress was reduced [48]. Oxidation resistance and conductivity of CF was enhanced by the continuous electroplating process of Ni which was found to be well-adhered to the CF [49].

With the goal of constructing hybrid joints for aerospace, automobile, and other technologies, in our studies, we have employed the CFP for strong adhesion of Metal/CFP/Metal and Metal/CFP/Polymer joints [7,8]. For example, the successful joining of Al/Ni-CF/Cu and Al/Ni-CF/Ti joints has been achieved by welding the half-lengths separately [23,24]. Later, a Ti/Ni-CF/Epoxy-CFRP joint fabricated by sputtering Ni on the CF before spot welding to Ti had a 100% increase in impact strength over Ti/Glue/Epoxy and Ti/Epoxy without CF [50]. The addition of the Ni plated CF of the Ti/Ni-CF/Epoxy-CFRP joint raised statistically the lowest impact value, *a*_s_ at *P*_f_ = 0 calculated by 3-parameter Weibull equation, to 2.20 kJm^−2^ from 0.35 and zero kJm^−2^ for Ti/Glue/Epoxy and Ti/Epoxy, respectively indicating an increase in safety and reliability. The Ni coating lowered or countered the effects of brittle carbides from being formed at the Ti-C interface while allowing improved interfacial contact between CF and metal half-length [50,51]. 

Since TPs are recyclable, we have recently developed a hybrid Ti/CFP/ABS joint by using an electron beam to spot weld Ti to a cross-weave CFP insert [12]. The remaining CFP half-length was then dipped in molten ABS. The Ti/CFP/ABS joint exhibited higher UTS than that of glue, Ti/Glue/ABS, or without glue, Ti/ABS [12], while simultaneously increasing strain at UTS (*ε*_T_).

However, joint strength is generally insufficient due to the encroachment of CF by hot molten metal along with brittle carbide formation. Therefore, we demonstrate employing CFP coated with Ni increases tensile mechanical properties for aluminum joints Al-ABS and Al-18-8. 

## 2. Materials and Methods

### 2.1. Sample Preparation

**Figure 2:**
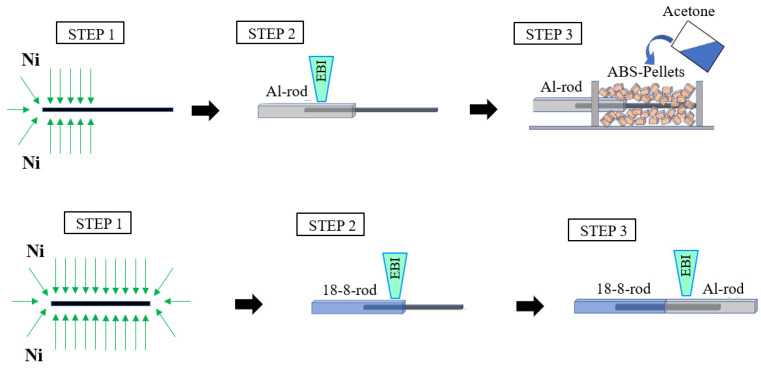
Schematic illustrations of joining process for Al/**Ni**-CFP/ABS and Al/**Ni**-CFP/18-8 joints.

Figure 2 shows schematic illustrations of the joining process of Al/**Ni**-CFP/ABS and Al/**Ni**-CFP/18-8 joints, respectively, as 3 basic steps.

### 2.2. Ni Coating Process (STEP 1)

STEP 1 is CFP cross weave (TORECA-M30SC, TORE; Tokyo, 6 µm) was coated by Ni interlayer surface skin. The inserted CFP is a cross-weave cloth that was embedded in the two joint half-lengths as shown in Figure 1. Ni-coating was prepared by DC-magnetron sputtering in the case of the Al/**Ni**-CFP/ABS joint [7]; and electro-plating with low cost in the case of the Al/**Ni**-CFP/18-8 joint [8]. In both cases, the CFs were observed to be covered perfectly with Ni [7,8]. Experimental conditions of both DC-magnetron sputtering [7] and electroplating [8] parameters are summarized in Table 1 and Table 2.

### 2.3. Al/**Ni**-CFP/ABS Joint (STEPS 2 and 3)

In joining the two materials, the higher MP half-length was formed first to the **Ni**-CFP (STEP 2) followed by the lower MP (or softening point) half-length (STEP 3). For the Al/**Ni**-CFP/ABS joint, STEP 2 is Al half-length was fabricated by spot welding Al rod to the CFP since MP of Al is higher than the softening temperature range of ABS [9,11,12,13]. To obtain maximum surface area contact and wetting within the intricate spaces in the weave, natural capillary phenomena was utilized before rapid solidification. STEP 3 is fabricating ABS half-length [7]. Here, ABS pellets were mixed with acetone prior to dipping the CFP, then heated to melting, followed by forming into the specimen shape, and drying [52]. ABS polymer solute: Acetone solvent molar ratio was 1:2. After rapid cooling for polymer amorphous structure, the Al/**Ni**-CFP/ABS samples were cut to size. In addition, samples of Al/Glue/ABS and Al/ABS were fabricated at ambient conditions. The glue used was LOCTITE 410, Henkel Japan, Tokyo, typically used for transportation equipment such as automobile parts, adhesion between metals, adhesion of ferritic magnets, and of rubber, metal and plastic in harsh environments [53]. Glue properties are main component of cyanoacrylate, use temperature range of −55 to 100 °C, and adhesion time of 60 to 120 sec [53].

### 2.4. Al/**Ni**-CFP/18-8 Joint (STEPS 2 and 3)

For Al/**Ni**-CFP/18-8 joint, STEP 2 is 18-8 rod was welded to the Ni-CFP first with a higher MP than Al [13]. STEP 3 is Al half-length was welded to the remaining Ni-CFP half-length [7].

The 99.3% pure Al (Nilaco Co., Ltd., Ginza, Tokyo, Japan), and 18-8 austenite stainless steel (18-8: SUS304 (Japan Industrial Standard JIS G 4304) [54] Nilaco Co., Ltd. Tokyo) were used. Welding the 18-8 and Al separately to the CFP by electron spot beam was employed to take advantage of fabricating a hybrid joint with different MPs [8]. Details of the spot beam apparatus (VA-8408, World Engineering Co., Ltd. Tokyo, Japan) are given in [8].

Al/CFP/18-8 joints without Ni were also fabricated. Al/18-8 without CFP could not be joined by spontaneous adhesion [8]. Sample dimensions in Figure 1 are listed in Table 3.

It is noted here spot welding process is an easy way to prepare single samples in the laboratory. However, for industrial applications, use of high frequency induction coil technique, generally used industrially for brazing, is useful to prepare larger numbers of CFP composites in a short time.

### 2.5. Tensile Testing and Characterization

An Autograph (Shimadzu Model AG-10TE, Tokyo, Japan) tensile tester was used to pull the joint specimens at a 1.0 mmmin^−1^ deformation rate at ambient conditions. Note since mechanical properties can be dependent on loading speed and temperature, employing dynamic mechanical thermal analysis (DMTA) under a wide temperature range with various tests such as bending, torsion and compression [55] would be extremely valuable for future research, but is beyond the scope of this study.

For the tensile tests, stress-strain data was taken as crosshead displacement and confirmed by video used to record the tests. Due to the heterogeneous deformation of the comparatively ductile half-length, for example, ABS in Al-ABS joints, and Al in Al-18-8 joints, traditional stress-strain curves could not be obtained [7,8]. Therefore, ultimate strength, σ_T_ (MPa) was taken as that from the nominal stress-strain (σ-ε) curves.

**Figure 3:**
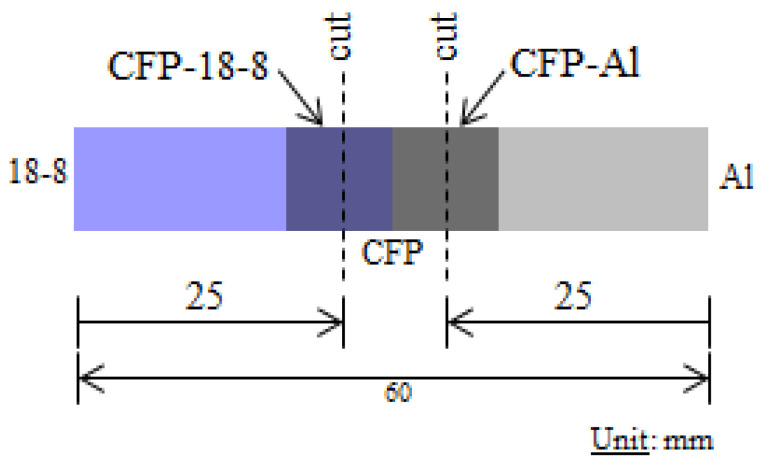
Cutting locations (dotted Lines) in Al-18-8 joints for XRD and EPMA analyses [8].

Electron probe micro-analyzer (EPMA-1610, 15 kV, 10 nA/Shimadzu, Kyoto, Japan) was utilized for elemental mappings on cross-sections to determine elemental migrations, and CF impingement or prevention [8]. In addition, X-ray diffraction (XRD) (Cu-K¡, MiniflexII, Rigaku, Tokyo, Japan) [8] was performed with 10^−3^ degs^−1^ scanning rate to obtain chemical elements and compounds from the lattice structures of diffraction peaks according to ICDD (International Centre for Diffraction Data). Cuts were made in the joint specimen with a diamond blade 25 mm from each end in the CFP-18-8, and CFP-Al sections as shown in Figure 3 [8].

## 3. Results and Discussion

### 3.1. Increase in UTS by Ni-CFP Plug

**Figure 4:**
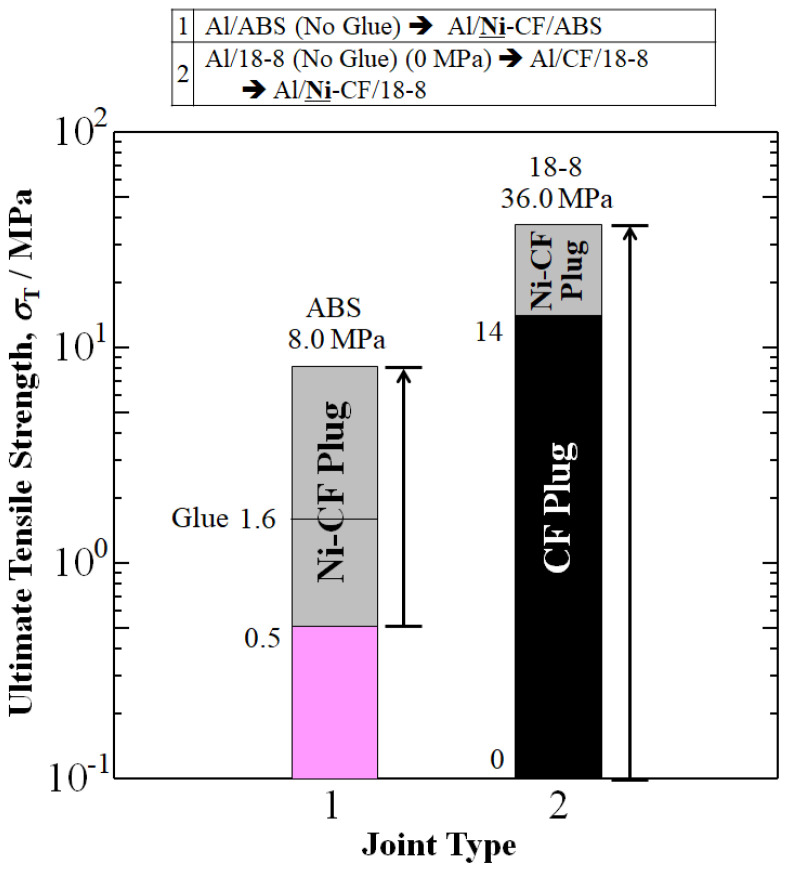
Improvements in UTS, *σ*_T_ (MPa) of aluminum joints Al/ABS and Al/18-8 by addition of Ni-plated CFP to make Al/**Ni**-CFP/ABS and Al/**Ni**-CFP/18-8 joints. Data is from: Shiraishi, Inui, Ishii, Matsumura, and Nishi (2014) [7]; and Tomizawa, Faudree, Kitahara, Takase, Matsumura, Jimbo, Salvia, and Nishi (2020) [8], for aluminum joints with ABS and 18-8, respectively.

Figure 4 shows an increase in reported experimental ultimate tensile strengths, *σ*_T_ (UTS) by Ni-CFP plug. UTS of Al/**Ni**-CFP/ABS, Al/Glue/ABS and Al/ABS joints were 8 MPa, 1.5 MPa and 0.5 MPa, respectively [7]. Employing the Ni-CFP in the Al/**Ni**-CFP/ABS joint raised *σ*_T_ 5 times over Al/Glue/ABS, and 16 times that of Al/ABS.

Without the CFP, a successful 18-8/Al joint could not be fabricated using conventional welding because of the formation of brittle metallic compounds [8]. However, implementation of the novel CFP assisted in generating maximum *σ*_T_ and its strain, *ε*_T_, thus confirming that Al and 18-8 could be joined together with CFP or Ni-CFP. The Al/CFP/18-8 joint achieved UTS of 14 MPa. However, Ni-coating on CFP in the Al/**Ni**-CFP/18-8 joint improved *σ*_T_, to 36 MPa, 2.6 times larger than that of Al/CFP/18-8 at 14 MPa.

Arrows in Figure 4 represent an increase in UTS from spontaneous adhesion of Al/ABS 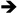 Al/**Ni-**CFP/ABS (16 times), and no adhesion of Al/18-8 to Al/**Ni**-CFP/18-8.

### 3.2. Rule of Mixtures Calculation for Corrected (Normalized) ^c^σ_T_ (^c^UTS) for CFRP Fractional Cross-Sectional Area

Rule of Mixtures is used to calculate corrected (normalized) UTS (^c^*σ*_T,JOINT_) for CFRP fractional cross-sectional area [51]:

σ_T,JOINT_ = Σ*n*_i_σ_T,i_ = *n*_CFRP_^c^*σ*_T_ + *n*_Al_*σ*s_T,Al-X_ (1)

where the subscript “T-JOINT” means experimental UTS in Figure 4, and in this case, components ‘*i*’ of *n*_i_ of “CFRP” and “Al” refer to their respective cross-sectional area fractions. The *σ*_T,Al-X_ is UTS of the joint without CFP. Solving for ^c^*σ*_T_ yields:

^c^*σ*_T_ = [*σ*_T,JOINT_ − *n*_Al_*σ*_T,Al-X_]/*n*_CFRP_ (2)

Here, *n*_CFRP_ and *n*_Al-X_ are approximated as 1/20 and 19/20 for Al-ABS joints, and 1/10 and 9/10 for Al/18-8 joints, respectively, in accordance with specimen geometry in Figure 1 and Table 3.

**Figure 5:**
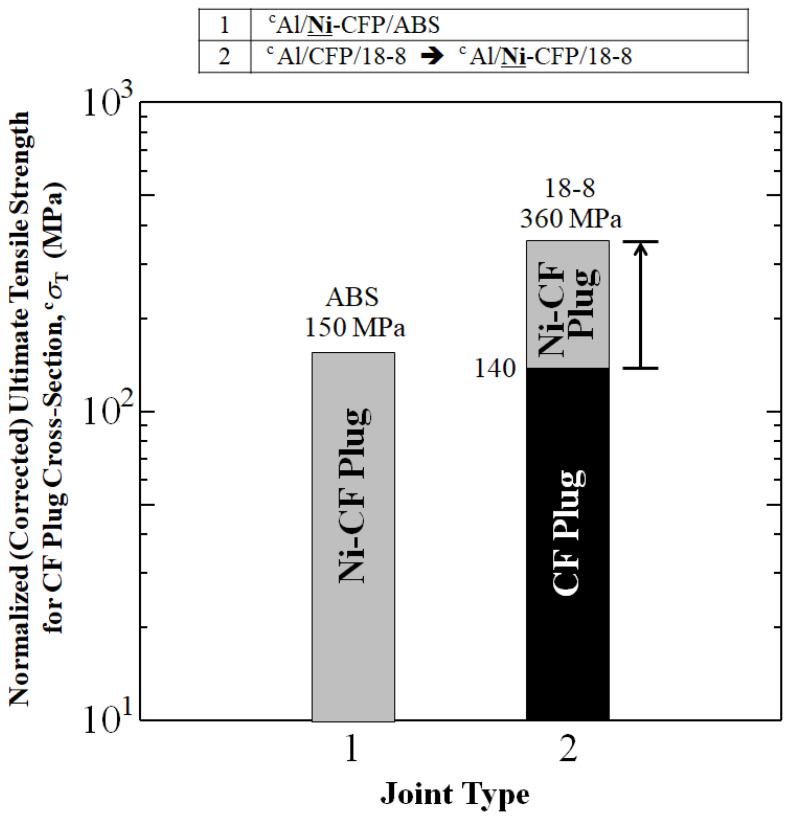
Enhancements in normalized UTS for CF plug cross-sections of aluminum joints Al/ABS and Al/18-8 by addition of Ni-plated CFP to make Al/**Ni**-CFP/ABS and Al/**Ni**-CFP/18-8 joints calculated from UTS values in [7,8]. Superscript ‘c’ designates normalized UTS.

Figure 5 shows when UTS is evaluated for CFRP cross-section, ^c^UTS of ^c^Al/**Ni**-CFP/ABS reaches 150.5 MPa, higher than that of the Metal/CFP/Metal joint without Ni-coating, ^c^Al/CFP/18-8 at 140 MPa. In the 18-8 joint, substantial 150% increase in ^c^UTS was achieved by coating the CFP with Ni. ^c^UTS was raised from 140 MPa for ^c^Al/CFP/18-8 to 360 MPa for the ^c^Al/**Ni**-CFP/18-8 joint.

### 3.3. Resistance Energy to Tensile Deformation, U_T_

Resistance energy to tensile deformation, *U*_T_ (kJm^−2^) is a measure of material toughness that can be used as an evaluation for potential load-bearing parts. When *X*_T_ is assumed to be 0.01 m-length of the CF-reinforced Al half-length of Al/**Ni-**CFP/18-8 with homogeneous uniaxial tensile deformation, the *U*_T_ is estimated by the integrated area under stress-strain curves reported in [7,8]. The *U*_T_ is generally calculated from zero strain to strain at maximum tensile strength, *σ*_T_ (*ε*_T_), and is approximately proportional to *X*_T_:

*U*_T_ = *X*_T_∫_0_*^ε^*^T^ σ d*ε*. (3)

**Figure 6:**
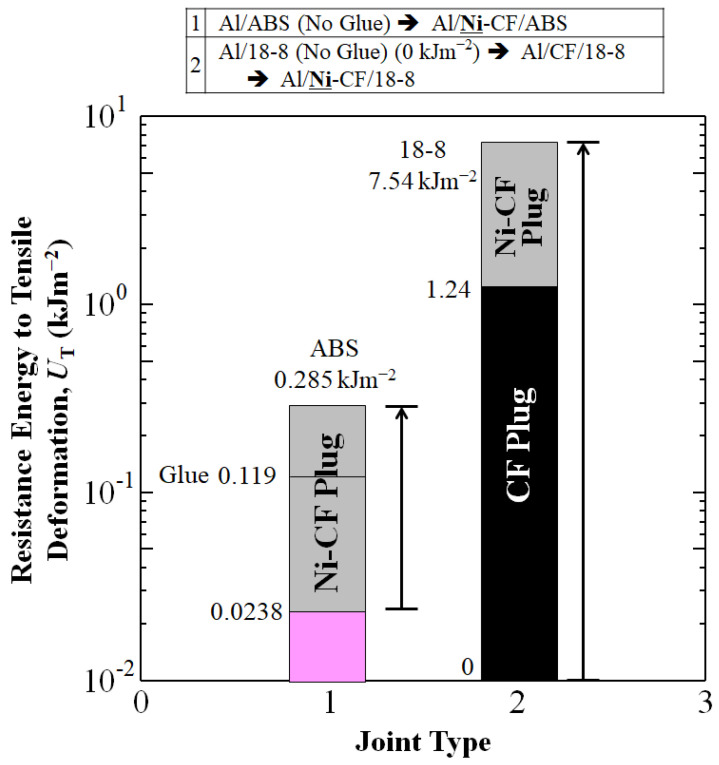
Improvements in resistance energy to tensile deformation, *U*_T_ (kJm^−2^) of aluminum joints Al/ABS and Al/18-8 by addition of Ni-plated CFP to make Al/**Ni**-CFP/ABS and Al/**Ni**-CFP/18-8 joints.

As shown in Figure 6, Ni-plating on CFP in the Al/**Ni-**CFP/ABS and Al/**Ni**-CFP/18-8 joints increase area under the stress-strain curve (*U*_T_) over that of Al/Glue/ABS, Al/ABS or Al/CF/18-8 joints.

When *X*_T_ is assumed to be 0.01 m-length between standard distance (0.02 m) of the CFs reinforced ABS half-length of Al/**Ni**-CFP/ABS, Al/Glue/ABS or Al/ABS with homogeneous uniaxial tensile deformation, *U*_T_ of Al/**Ni**-CFP/ABS joint (0.285 kJm^−2^) was apparently improved, about 12.0 and 2.40 times larger than that of Al/Glue/ABS (0.119 kJm^−2^) and Al/ABS (0.0238 kJm^−2^) joints as shown in Figure 6. *U*_T_ of Al/**Ni**–CFP/18-8 joint (7.54 kJm^−2^) was also apparently improved, about 6.08 and 26.5 times larger than that of Al/CFP/18-8 (1.24 kJm^−2^) and Al/**Ni**-CFP/ABS (0.285 kJm^−2^) joints. Calculated *U*_T_ values, along with *s*_T_ and *e* at *s*_T_ are summarized in Table 4.

### 3.4. Stress-Strain Curves and Specific Tensile Strength, ^s^s_T_

**Figure 7:**
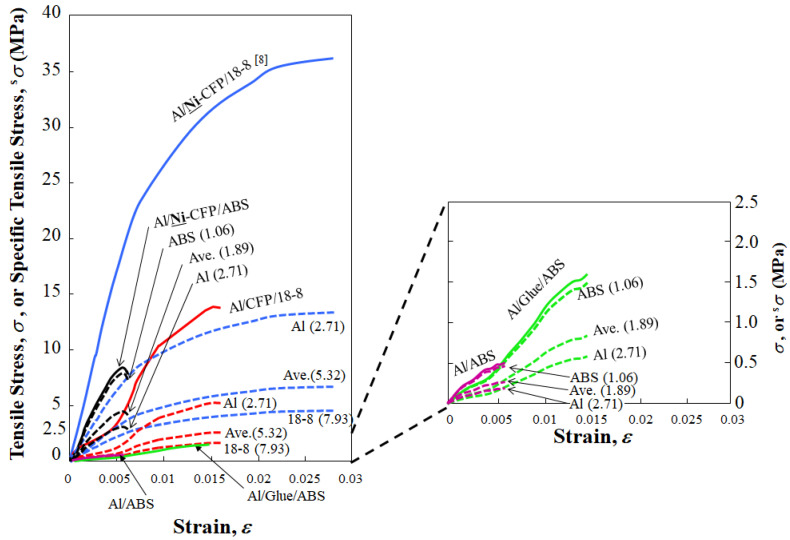
Specific tensile stress-strain (^s^*σ*–*ε*) curves (dotted lines) for Al/**Ni**-CFP/ABS (black), Al/Glue/ABS (green), Al/ABS (purple), Al/**Ni**-CFP/18-8 (blue) and Al/CFP/18-8 (red). Specific gravity values (*ρ*) used are indicated: Al (2.71), 18-8 (7.93), ABS (1.06) along with mean values (*ρ*_mean_) of Al/18-8 (5.32) and Al/ABS (1.89). The The (^s^*σ*–*ε*) curves are calculated from tensile stress-strain (*σ*–*ε*) curves (solid lines, same colors) taken from: Shiraishi, Inui, Ishii, Matsumura, and Nishi (2014) [7]; and Tomizawa, Faudree, Kitahara, Takase, Matsumura, Jimbo, Salvia, and Nishi (2020) [8], for aluminum joints with ABS and 18-8, respectively.

Figure 7 shows tensile stress-strain (*σ*–*ε*) curves (solid lines) of the data from Figure 4 reported in [7,8]. Although the strain at tensile strength (*ε*_T_) of the Al/**Ni**-CFP/ABS joint (0.006) is equal to that of Al/ABS (0.006), that of Al/Glue/ABS (0.015) is 2.5 times higher than that of Al/ABS and Al/**Ni**-CFP/ABS (0.006) from increased ductility by the glue. Ni-coating on CFP in the Al/**Ni**-CFP/18-8 joint improves *σ*_T_, as well as its strain (ε_T_) over that of 18-8/CF/Al. Namely, *σ*_T_ and *ε*_T_ of Al/**Ni**–CFP/18-8 joint (36 MPa, 0.028) are 2.6 and 1.8 times larger than that of Al/CFP/18-8 joints (14 MPa, 0.016), respectively. Furthermore, the *σ*_T_ and *ε*_T_ of Al/**Ni**–CFP/18-8 joint (36 MPa, 0.028) are 4.5 and 4.7 times larger than those of Al/CFP/ABS (8 MPa, 0.006), respectively. The effect of conversion from light ABS polymer to heavy 18-8 stainless steel alloy increased *σ*_T_ and *ε*_T_, (28 MPa, 0.022), from Al/**Ni**-CFP/ABS joint (8 MPa, 0.006) to Al/**Ni**–CFP/18-8 joint (36 MPa, 0.028).

To evaluate materials taking into account the tradeoff of strength vs. lightness (strength/weight ratio) with consideration for fuel conservation, the parameter of the specific tensile strength (^s^*σ*_T_) is calculated using the specific gravity, *ρ* of joint components:

^s^*σ*_T_ = *σ*_T_ *ρ*^−1^ (4)

**Figure 8:**
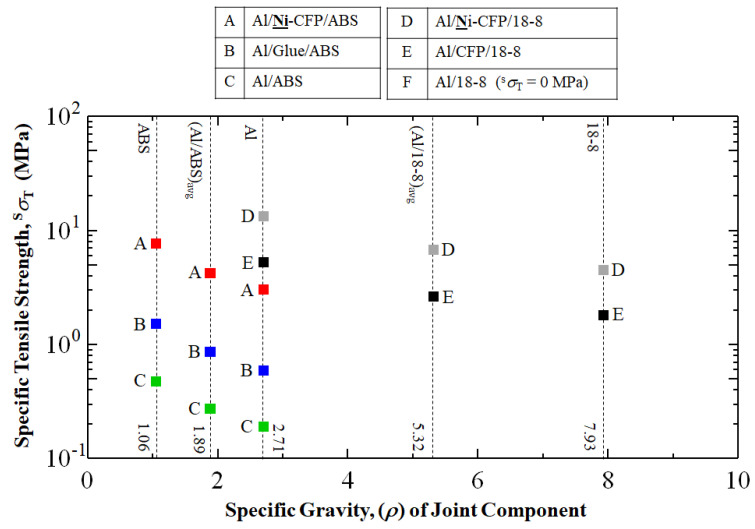
Specific tensile strength, ^s^*σ*_T_ (MPa) calculated according to density (gcm^−3^) (specific gravity) of joint component. The square data points of colors red, blue, green, gray and black represent Al/**Ni**-CFP/ABS, Al/Glue/ABS, Al/ABS, Al/**Ni**-CFP/18-8, and Al/CFP/18-8 joints, respectively. Al/18-8 joint without CFP could not be fabricated due to brittle carbide formation.

Figure 8 compares specific tensile stress-strain (^s^*σ*–*ε*) curves (dotted lines) along with specific tensile strengths, ^s^*σ*_T_ for Al/**Ni**-CFP/18-8, Al/CFP/18-8, Al/**Ni**-CFP/ABS, Al/Glue/ABS, and Al/ABS according to *ρ* of material contained in the joints. Density values are used for specific gravity, as indicated [10,56].

As expected, Figure 8 shows the lighter specific gravity (*ρ*) used, the higher ^s^*σ*_T_ is calculated to be. Interestingly, when basing ^s^σ_T_ on *ρ*_mean_ (*ρ*_Al/ABS,avg_ = 1.89), Figure 8 shows the Al/**Ni**-CFP/ABS joint with TP polymer and Ni-CFP has ^s^σ_T_ of 4.2 MPa (red square), 160% higher than that of Al/CFP/18-8 with uncoated CFP based on *ρ*_mean_ (*ρ*_Al/18-8,avg_ = 5.32) at 2.6 MPa (black square). Therefore, when basing ^s^σ_T_ on *ρ*_mean_, Ni coating to CFP increases strength to weight ratio of the TP containing joint Al/**Ni**-CFP/ABS over that of the Metal-Metal joint of Al/CFP/18-8.

As expected, when using specific gravity of Al *ρ*(Al) = 2.71 as the basis, the hierarchy of ^s^σ_T_ follows the tensile strength (σ_T_) hierarchy highest to lowest of σ_T_ in Figure 4 of Al/**Ni**-CFP/18-8 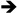 Al/CFP/18-8 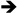 Al/**Ni**-CFP/ABS 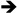 Al/Glue/ABS 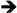 Al/ABS.

However, Figure 8 shows when basing ^s^σ_T_ on the “material other than Al”, that is, *ρ*_(18-8)_ = 7.93 or *ρ*_(ABS)_ = 1.06, the Al/**Ni**-CFP/ABS joint can be far superior with ^s^σ_T_ of 7.6 MPa, over those of Metal/Metal including Al/**Ni**-CFP/18-8 at 4.5 MPa, Al/CFP/18-8 at 1.8 MPa, along with Al/Glue/ABS at 1.5 MPa, and Al/ABS at 0.47 MPa. But considering specific gravity, the conversion merit should be the opposite effect. The specific gravity selected for ^s^σ_T_ calculation of a joint will depend on the specific aerospace or other application, relative masses and geometries of parts, stress distributions, temperature, gravity of Earth or other heavenly body, and if in an atmosphere, thrust, lift, and drag of the entire vehicle, among other factors.

### 3.5. Specific Resistance Energy to Tensile Deformation

Specific resistance to tensile deformation (^s^*U*_T_) is a measure of material toughness and can be useful for material analyses. Based on Equation (1) and the integrated area under the specific stress-strain curves in Figure 7, ^s^*U*_T_ is also calculated using specific gravity, *ρ*:

^s^*U*_T_ = *U*_T_*ρ*
^−1^ (5)

**Figure 9:**
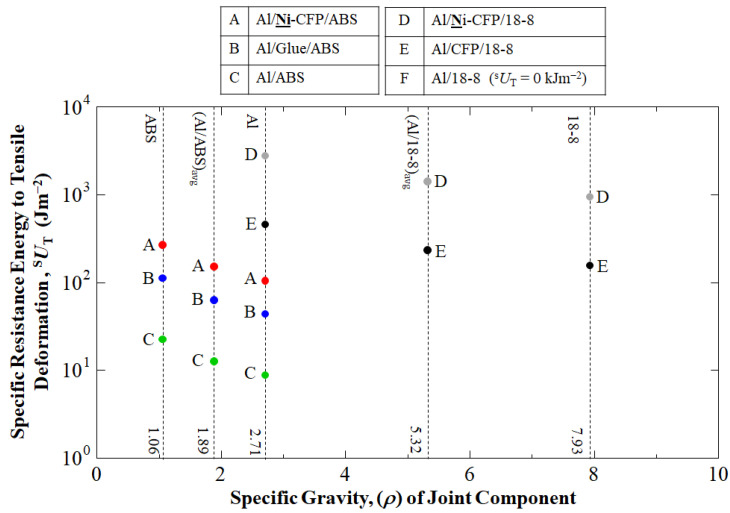
Specific resistance energy to tensile deformation, ^s^*U*_T_ (Jm^−2^) calculated according to density (gcm^−3^) (specific gravity) of joint component.

Figure 9 shows as expected, ^s^*U*_T_ of the Al/**Ni**-CF/18-8 joint is larger than that of Al/**Ni**-CFP/ABS. The ^s^*U*_T_ of the Al/**Ni**–CFP/18-8 (1420, 2780 and 951 Jm^−2^) are 9.40, 26.5 and 3.54 larger than that of Al/**Ni**-CFP/ABS (151, 105 and 269 Jm^−2^), respectively.

Although the ^s^*U*_T_ results can be different than that of ^s^σ_T_, both Al/**Ni**-CFP/ABS and 18-8/**Ni**-CF/Al joints should be aimed to be utilized for their most adaptable aerospace or other applications. The circular data points of colors red, blue, green, gray and black represent Al/**Ni**-CFP/ABS, Al/Glue/ABS, Al/ABS, Al/**Ni**-CFP/18-8, and Al/CFP/18-8 joints, respectively. Al/18-8 joint without CFP could not be fabricated due to brittle carbide formation. 

### 3.6. Fracture Mechanisms of Al-ABS Joints

**Figure 10:**
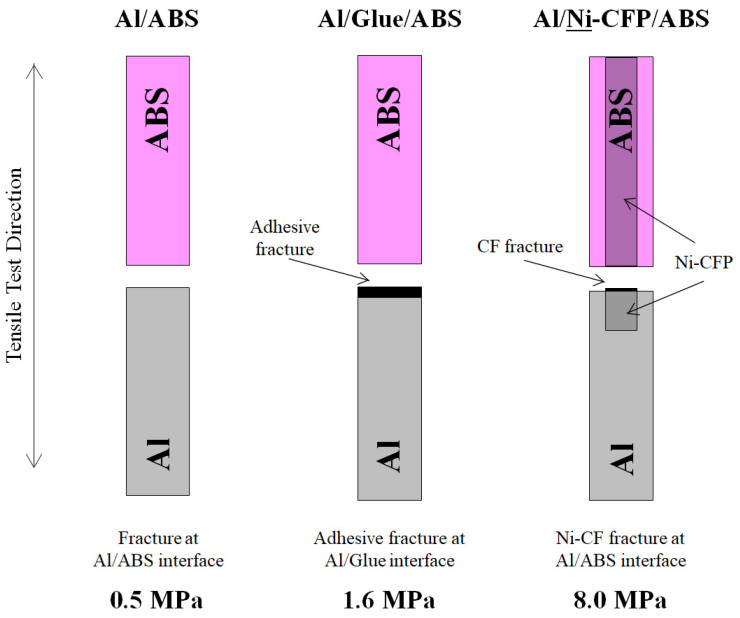
Schematic of fractured Al-ABS joint specimens adapted from Shiraishi et al. (2014) [7]. UTS values (MPa) are indicated. Ni-CFP is indicated for clarity although not visible on specimen surface (see Figure 1 and Table 3).

Figure 10 adapted from Shiraishi et al. (2014) [7] shows fracture types and location for Al-ABS joints. The Al/ABS joint exhibited adhesive type fracture between Al and ABS half-lengths. Here “adhesive” refers to the crack being between the ABS and Al rather than propagating within either. The Al/Glue/ABS joint also exhibited adhesive type fracture since the main crack was between the glue and ABS. The adhesive fractures led to lower UTS values.

For the Al/Ni-CFP/ABS joint, although the CFP has not been a traditional “adhesive” to join two surfaces, the fracture type is rendered here as “cohesive” since the fracture occurred within the CFP, remaining in both half-lengths.

UTS was increased from 0.5 MPa for Al/ABS to 1.6 MPa for Al/CFP/ABS, and further to 8.0 MPa for Al/Ni-CFP/ABS by the strong Ni-CFP taking on the load and exhibiting fiber fracture. Although reported UTS of CF itself is high, ranging from about 2.5 to 6 GPa [57], fiber breakage occurs due to: being less ductile than ABS or Al, small 6 mm diameter [7], with decent coupling to Al and ABS to prevent fiber pull-out.

For all Al-ABS joints in Figure 10, main cracks were straight across specimen width at the Al/ABS interface perpendicular to tensile test direction.

### 3.7. Fracture Mechanisms of Al-18-8 Joints

**Figure 11:**
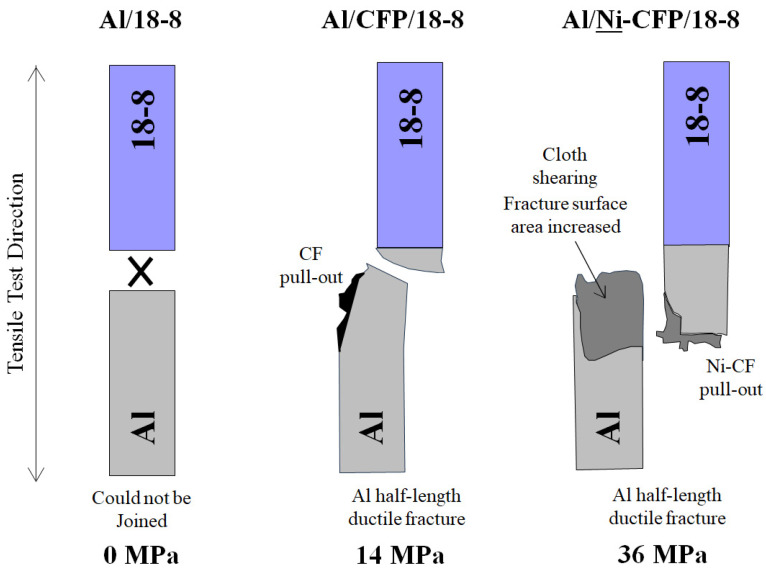
Schematic of Al-18-8 joint specimens adapted from photos in Tomizawa et al. (2020) [8]. Al and 18-8 were nonjoinable by spot welding, hence UTS was zero. However, utilization of either CFP or Ni-CFP allowed Al and 18-8 to be successfully joined [8]. UTS values (MPa) are shown. CFP and Ni-CFP are not indicated (as in Figure 10) to clearly show the fracture type (see Figure 1 and Table 3).

Figure 11 adapted from photos in Tomizawa et al. (2020) shows the Al/**Ni**-CFP/18-8 joint exhibited significantly expanded fracture surface area over Al/CFP/18-8 to raise UTS from 14 to 36 MPa. In the Al half-length of the Al/**Ni**-CFP/18-8 joint, there was ductile fracture, CFP cloth shearing about 10 mm along the length of the specimen, with CF pull-out and single fiber isolation exemplifying expanded fracture area and fracture energy absorption [8]. CF breakage was also observed.

Fracture type of the Al/**Ni**-CFP/18-8 joint is rendered here as “cohesive” since it occurred within the intricacies of the Ni-CFP itself penetrated 10 mm deep into the Al, rather than at the Al/18-8 interface, or at CFP edge/Al matrix transition zone across specimen width. The advantage of CFP is it adheres by broad surface area of thin (6 mm) CFs for enhanced adhesion not only at the interface, but 10 mm deep into each half-length, with the Ni-coating improving CF-Al adhesion.

In contrast, Figure 11 shows Al/CFP/18-8 joint without Ni had much smaller fracture surface area, spanning approximately across specimen width. CF cloth shearing was not observed. Ductile fracture occurred in the Al half -length near the Al/18-8 interface accompanied by CF breakage and slight CF pull-out. No or little single fiber isolation was observed [8]. Again, fracture type would be classified here as “cohesive” since part of CFP remains in both Al and 18-8 half-lengths. The lower UTS is attributed to poorer Al-CF contact with brittle carbides, and impingement of CF by heat of welding.

In both Al/**Ni**-CFP/18-8 and Al/CFP/18-8, the 18-8 half-length was unaffected. This is due to typical 18-8 stainless steel (304) having higher tensile modulus of 193 GPa compared to that of Al (3003-H14) at 70 GPa [8].

In sum, employing the CFP makes joining of Al and 18-8 possible, while coating Ni to the CFs prior to spot welding increases UTS further, over 2 times that without Ni.

### 3.8. Metallographic Processes of Al Half-Length: XRD and EPMA Data

SEM, XRD, and EPMA data have already been reported by Shiraishi et al. (2014) [7] and Tomizawa et al. (2020) [8] but will be summarized in here for convenience. As for SEM, photographs of Ni-CFP showed remarkable coverage of Ni film on the CFs for enhanced adhesion and are given in [7,8].

**Figure 12:**
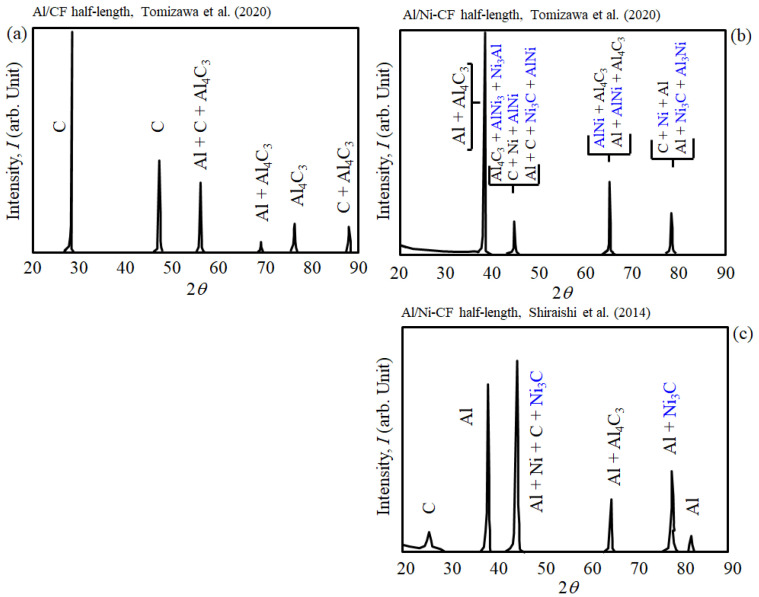
Summary of XRD analysis data of welded: (**a**) Al/CF and (**b**,**c**) Al/**Ni**-CF half-lengths. Ni compounds indicated in blue. Adapted from Tomizawa et al. (2020) [8]; and Shiraishi et al. (2014) [7].

**Figure 13:**
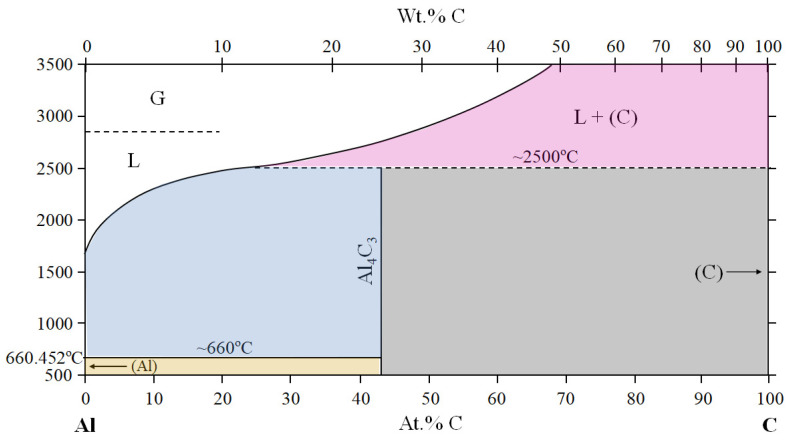
Al-C phase diagram adapted from Dabouz et al. (2019) [58].

Figure 12 shows a summary of X-ray diffraction (XRD) data of Al/CF and Al/**Ni**-CFP half-lengths from Tomizawa et al. (2020) [8]; and Shiraishi et al. (2014) [7]. The Ni-coating appears to lower or counter effects of carbide formation. Figure 12a shows without Ni coating, brittle Al_4_C_3_ formation occurred in the Al/CFP half-length [7] as evidenced by four peaks [8] resulting in the lower UTS. The Al-C phase diagram in Figure 13 shows when Al is heated and melted by the spot welding, there will be travel along the liquidus forming Al_4_C_3_ in the vicinity of the CFs [58].

In contrast, for Al/**Ni**-CFP half-length, Figure 12b,c show despite Al_4_C_3_ being generated, formation of Ni-Al intermetallic compounds Ni_3_Al, AlNi, Ni_3_C, Al_3_Ni, and the metal Ni [7,8] (indicated in blue) are also detected within the same diffraction angles, 2*q*. This is clear evidence the Ni coating acts as a buffer [8] raising UTS.

**Figure 14:**
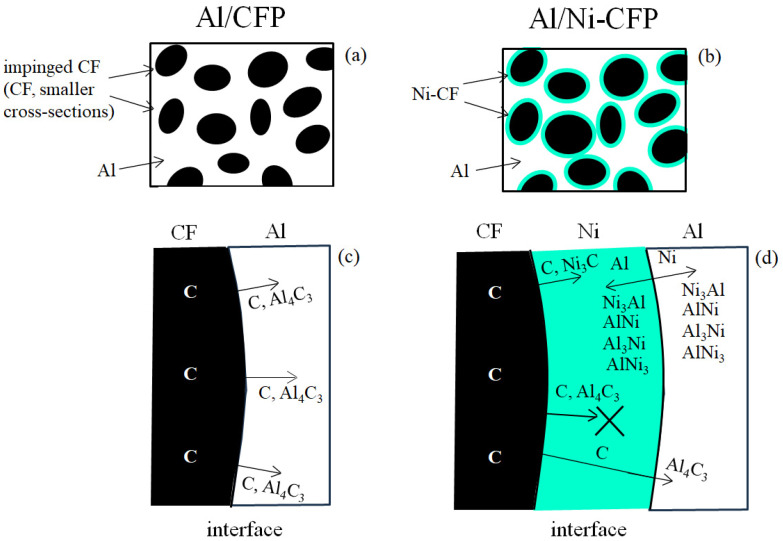
Summary from EPMA and XRD analyses of Al half-length in [7,8]. For Al/CFP and Al/Ni-CFP half-lengths, respectively, (**a**,**b**) are that of EPMA, (**c**,**d**) are that of XRD.

Figure 14 shows a graphical summary of EPMA data from Tomizawa et al. (2020) [8]; and Shiraishi et al. (2014) [7]. Figure 14a,b depict CF cross-sectional areas are smaller in the Al/CF half-length than that of Al/**Ni**-CFP. The impingement from spot welding directly to the CF is prevented by the Ni coating. Figure 14b shows larger CF cross-sections are maintained encompassed by their Ni coatings (blue color).

Figure 14c,d illustrates action by the Ni coating at the CF-Al interface. Diffusion directions are indicated by arrows. EPMA confirmed the brittle carbide Al_4_C_3_ formation in the Al/CFP half-length [8]: Figure 14c shows without the Ni coating, C atoms and Al_4_C_3_ diffuse into the Al. On the other hand, in the Al/**Ni**-CFP half-length in Figure 14d, three main strengthening processes are present. First, the Ni acts as diffusion barrier. Most Al_4_C_3_ diffusion into the Al is prevented as indicated by ‘X’. The smaller amount that does diffuse into the Al was reported to diffuse at shorter distances (arrow) [8]. Secondly, Ni diffusion layer could be observed at the Al/CF interface zone [8]. Mutual diffusion of Ni-Al compounds Ni_3_Al, AlNi, Al_3_Ni, and AlNi_3_ and Ni and Al atoms occurs across the Ni/Al interface strengthening the bond to take on more of the load during tensile deformation resulting in the shear fracture. Thirdly, as mentioned earlier, the Ni coating prevents CF impingement damage. As for within the CF, metallic elements could not be precisely detected: EPMA analysis showed Ni and Al atoms were not present in CF [7].

### 3.9. Metallographic Processes of 18-8 Half-Length: XRD and EPMA Data

Ni compounds are indicated in blue. Adapted from Tomizawa et al. (2020) [8].

Since the MP of 18-8 is much higher than that of Al, damage to CF by impingement during welding is a more serious concern. Although the 18-8 half-length escapes damage in the Al/CFP/18-8 joints due to its higher ductility and lower UTS than Al, EPMA results have shown reduced CF diameters by impingement from molten 18-8 [8]. Therefore, for maximum safety, the 18-8 half-length is also coated with Ni.

**Figure 15:**
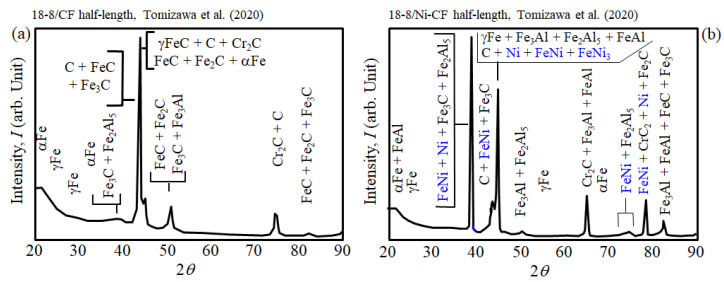
Summary of XRD analysis data of welded: (**a**) 18-8/CF and (**b**) 18-8/Ni-CF half-lengths.

Figure 15a shows without Ni, XRD analysis of the 18-8/CF half-length of the Al/CFP/18-8 joint detected carbides of FeC, Fe_2_C, Fe_3_C, Cr_2_C and C [8]. EPMA mapping results showed αFe crystal grains 10 to 30 mm in size that nucleated and grew between liquidus and solidus into the residual molten steel [8]. γFe grains were generated at the solidus. Carbon (C) and Cr element concentrations were higher in γFe than in αFe, and Ni concentration was higher in αFe than in γFe [8].

On the other hand, Figure 15b shows in the Al/**Ni**-CFP/18-8 joint, in addition to carbides, Ni compounds were detected in the 18-8 half-length by XRD including FeNi and FeNi_3_ [8].

**Figure 16:**
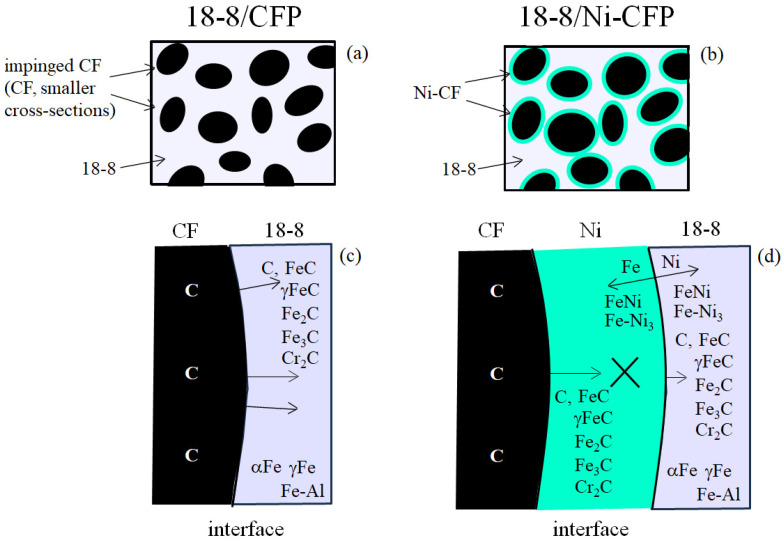
Summary from EPMA and XRD analysis of 18-8 half-length in [8]. For 18-8/CFP and 18-8/Ni-CFP half-lengths, respectively, (**a**,**b**) are that of EPMA, (**c**,**d**) are that of XRD.

A graphical summary of EPMA data of the 18-8/CF half-length from Tomizawa et al. (2020) [8] is given in Figure 16a,b, where (a) shows smaller CF cross-sections by impingement by the high 18-8 welding temperature, and (b) shows larger CF cross-sections maintained by the Ni coating (blue color).

Figure 16c,d illustrate EPMA data of action by the Ni coating at the CF-18-8 interface with diffusion directions (arrows). Figure 16c shows without the Ni coating, C atoms and Fe-C carbides are present diffusing into the 18-8. On the other hand, Figure 16b shows Ni coating apparently prevents or decreases excess carbides from diffusing into the 18-8 indicated by ‘X’. Similar to the Al/**Ni**-CFP half-length, a diffusion barrier is observed, formed of FeNi with FeNi_3_, mutually diffusing into the Ni and 18-8, respectively.

Note Fe-Al compounds were detected, probably transferring easily from the Al half-length during the intense heat from the 18-8 welding temperature. Most importantly, in the 18-8/CF half-length, the Ni coating prevents impingement damage to the CF.

**Figure 17:**
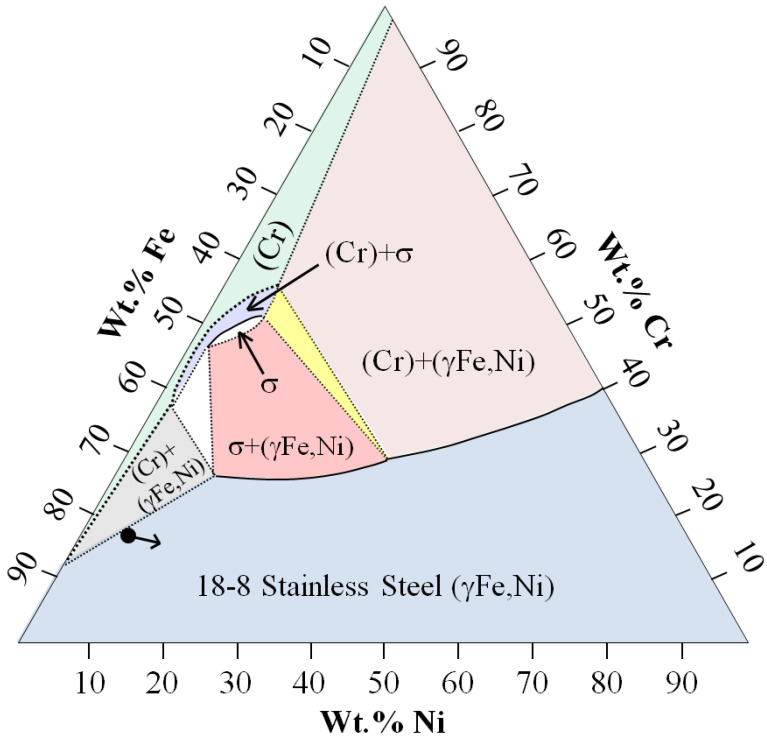
18-8 (Fe-Cr-Ni) Tertiary phase diagram adapted from Nam, Kim and Kim (2017) [59]. Apparent wt.% composition change by the Ni coating is indicated by the arrow.

To characterize the metallographic changes, a quaternary Fe-Cr-Ni-C phase diagram would be of interest but is beyond the scope of this study. Therefore, Figure 17 illustrates the effect of Ni addition on composition (arrow) with the tertiary 18-8 (Fe-Cr-Ni) diagram [59] by the intersection of wt.% lines. Near the CFs, as the Ni coating diffuses into the 18-8 and increases in concentration, wt.% of Fe and Cr decrease with an overall relative concentration in the 18-8 stainless steel (γFe,Ni) phase.

## 4. Conclusions

Al is a lightweight material that is frequently joined with other materials to make hybrid joints for numerous applications such as commercial aircraft, space vehicles, automobiles and sports equipment to name a few. Therefore, strong hybrid joints with Al are always highly sought after. New types of hybrid Al joints connected by a novel Ni-coated carbon fiber plug (CFP) cross weave: Al-acrylonitrile butadiene styrene (ABS) carbon fiber reinforced thermoplastic polymer (CFRTP) designated Al/**Ni**-CFP/ABS, and Al-18-8 Stainless steel, Al/**Ni**-CFP/18-8 not before seen in the literature have been fabricated. The goal is to take advantage of extremely high ~6 mm CF surface area for high adhesion, to enhance safety level of aircraft and other parts. This is without fasteners, chemical treatment, or glue. Rapid melting followed by rapid solidification was found to allow the molten Al and 18-8 to flow intricately into the CFP weave for significant enhancement over that without CFP. Tensile properties of UTS, resistance energy to tensile deformation (*U*_T_) and specific tensile strength (^s^*σ*_T_) were enhanced by the CFP. This research is still in the development phase, hence carefulness is highly recommended if employing for practical use.

## Figures and Tables

**Table 1:** DC-magnetron sputtering parameters. (Ni) [7].

Parameter	Value
Leak rate (Pa·m^3^·s^−1^)	8 × 10^−6^ to 1 × 10^−4^
Residual gas pressure (Pa)	below 1.5 × 10^−3^
Ar gas sputtering pressure (Pa)	5.0 ×10^−1^
Sputtering potential (V)	300
Sputtering current (A)	0.7
Deposition rate (µm·h^−1^)	30

**Table 2:** Electro-plating parameters. (Ni) [8].

Parameter	Value
Current (A)	1.5
Voltage (V)	7.0
Electro-plating time (min)	30
Temperature (K)	298
Water solution: 400 mL with 12 g boric acid; 100 g nickel sulfate; 18 g nickel chloride	

**Table 3:** Dimensions of tensile test joint samples.

JOINT (Units in mm)	*l*	*w*	*t*	CFP l	CFP w	CFP th	CFP in Al	CFP in ABS	CFP in 18-8
Al/Ni-CFP/ABS-CFRTP	70	10	3	42	5	0.3	7	35	-
Al/Ni-CFP/18-8	60	10	3	20	10	0.3	10	-	10
Al/ABS	70	10	3				-	-	-
Al/Glue/ABS	70	10	3				-	-	-

**Table 4:** Resistance energy to tensile deformation (*U*_T_), along with reported tensile strength (*σ*_T_) in Figure 4 and Figure 6 and its strain (*ε*_T_) [7,8].

Joint	*U*_T_ (kJm^−2^)	*σ*_T_ (MPa)	*ε*_T_
[Al/Ni-CFP/ABS]	0.285	8	0.006
[Al/Glue/ABS]	0.119	1.6	0.015
[Al/ABS]	0.0238	0.5	0.006
[Al/Ni-CFP/18-8]	7.54	36	0.028
[Al/CFP/18-8]	1.24	14	0.016
[Al/18-8]	0	0	0

## Data Availability

Data is available upon request from the corresponding author. There was no obligation to make the data publicly available during the course of this project.
